# Lack of Correlation between In Vitro and In Vivo Studies on the Inhibitory Effects of (‒)-Sophoranone on CYP2C9 Is Attributable to Low Oral Absorption and Extensive Plasma Protein Binding of (‒)-Sophoranone

**DOI:** 10.3390/pharmaceutics12040328

**Published:** 2020-04-07

**Authors:** Yu Fen Zheng, Soo Hyeon Bae, Zhouchi Huang, Soon Uk Chae, Seong Jun Jo, Hyung Joon Shim, Chae Bin Lee, Doyun Kim, Hunseung Yoo, Soo Kyung Bae

**Affiliations:** 1 School of Basic Medicine and Clinical Pharmacy, China Pharmaceutical University, 639 Longmian Road, Jiangning District, Nanjing 211198, China; 1020172557@cpu.edu.cn; 2 Q-fitter, Inc., Seoul 06578, Korea; sh.bae@qfitter.com; 3 College of Pharmacy and Integrated Research Institute of Pharmaceutical Sciences, The Catholic University, Korea, Bucheon 14662, Korea; hzc0826@catholic.ac.kr (Z.H.); zldtnseoz@naver.com (S.U.C.); sungjun6734@naver.com (S.J.J.); tony6533@naver.com (H.J.S.); aribri727@catholic.ac.kr (C.B.L.); doyun325@naver.com (D.K.); 4 Life Science R&D Center, SK Chemicals, 310 Pangyo-ro, Sungnam 13494, Korea; hs.yoo@sk.com

**Keywords:** (‒)-sophoranone, CYP2C9, potent inhibition, in vitro, in vivo, drug interaction, low permeability, high plasma protein binding

## Abstract

(‒)-Sophoranone (SPN) is a bioactive component of *Sophora tonkinensis* with various pharmacological activities. This study aims to evaluate its in vitro and in vivo inhibitory potential against the nine major CYP enzymes. Of the nine tested CYPs, it exerted the strongest inhibitory effect on CYP2C9-mediated tolbutamide 4-hydroxylation with the lowest IC_50_ (*K*_i_) value of 0.966 ± 0.149 μM (0.503 ± 0.0383 μM), in a competitive manner. Additionally, it strongly inhibited other CYP2C9-catalyzed diclofenac 4′-hydroxylation and losartan oxidation activities. Upon 30 min pre-incubation of human liver microsomes with SPN in the presence of NADPH, no obvious shift in IC_50_ was observed, suggesting that SPN is not a time-dependent inactivator of the nine CYPs. However, oral co-administration of SPN had no significant effect on the pharmacokinetics of diclofenac and 4′-hydroxydiclofenac in rats. Overall, SPN is a potent inhibitor of CYP2C9 in vitro but not in vivo. The very low permeability of SPN in Caco-2 cells (P_app_ value of 0.115 × 10^−6^ cm/s), which suggests poor absorption in vivo, and its high degree of plasma protein binding (>99.9%) may lead to the lack of in vitro–in vivo correlation. These findings will be helpful for the safe and effective clinical use of SPN.

## 1. Introduction

**Figure 1:**
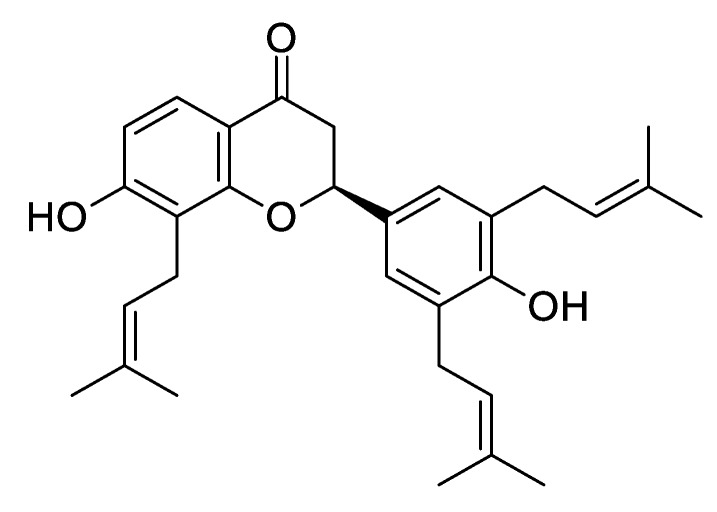
Chemical structure of (‒)-sophoranone (SPN).

(‒)-Sophoranone (SPN; Figure 1), a major bioactive flavonoid isolated from the roots of *Sophora tonkinensis*, is used in traditional Chinese medicine for the treatment of acute pharyngolaryngeal infections and sore throat [1,2,3]. It exhibits anti-inflammatory effects by inhibiting nitric oxide production in macrophages [4] and 5-lipoxygenase activity [3]. Several studies have also demonstrated its other biological activities, such as anti-cancer [5], anti-diabetic diabetic [6], and immunomodulatory [7] activities. In our previous study, after orally administering 12.9 mg/kg SPN to rats, the maximum plasma concentration (C_max_) was approximately 13.1 ng/mL at 60 min [8]. Thus, although conclusive results are lacking, SPN is likely to be a promising drug candidate.

Drug–drug interactions can increase the likelihood of treatment failure or the frequency and severity of adverse events [9]. Thus, drug–drug interaction assessment is a critical component of new drug discovery and development as well as clinical practice [9,10]. The majority of known drug interactions occur because of inhibition of drug-metabolizing enzymes [11,12,13]. Among all drug-metabolizing enzymes, the cytochrome P450 (CYP) superfamily plays an important role in the oxidation of almost 90% of currently used drugs [14]. Among at least 57 human cytochrome P450 enzymes identified to date, 9 hepatic P450 enzymes (CYP1A2, 2A6, 2B6, 2C8, 2C9, 2C19, 2D6, 2E1, and 3A4) have shown to play predominant roles in the metabolism of drugs and other xenobiotics [12]. Therefore, the inhibitory potential of SPN on the nine major CYP enzymes should also be investigated. There are a few reports on the in vitro and in vivo inhibitory effects of SPN on CYP enzymes. In rats, oral administration of 5 g/kg *S. tonkinensis* extract over 14 days was found to increase the plasma concentrations of metoprolol, omeprazole, and bupropion. This might be attributed to the inhibition of the activities of rat CYP enzymes, CYP2D6, CYP2C19, and CYP2B6 [15]. However, these results could not directly reflect the in vivo inhibitory potential of SPN on CYP enzymes due to multiple components of the extract. Several flavonoids, including SPN, have been found to inhibit CYP3A4-mediated reactions in vitro [16].

However, currently, there is limited information about SPN’s in vitro inhibitory potentials, especially on the other eight CYP enzymes, thereby warranting further in vitro and in vivo investigations to improve our understanding of drug interactions with SPN. Using human liver microsomes in this study, we evaluated SPN’s potential to inhibit CYP1A2, CYP2A6, CYP2B6, CYP2C8, CYP2C9, CYP2C19, CYP2D6, CYP2E1, and CYP3A4 in a reversible and time-dependent manner. We report herein that SPN is a potent inhibitor of CYP2C9 in vitro but not in vivo. To explain this lack of correlation between in vitro and in vivo results, we performed plasma protein binding of SPN and permeability test using Caco-2 cells.

## 2. Materials and Methods

### 2.1. Chemicals and Reagents

Pooled human liver microsomes from 150 donors (75 males; 75 females) were purchased from Corning Life Sciences (Woburn, MA, USA), and (‒)-sophoranone (99.7% purity; SPN) was supplied by SK Chemicals Ltd. (Sungnam, Gyeonggi-do, Korea). β-Nicotinamide adenine dinucleotide phosphate disodium salt (NADP), glucose 6-phosphate disodium salt hydrate, glucose 6-phosphate dehydrogenase, MgCl_2_, and all chemicals including the specific substrates, its metabolites, and well-known inhibitors of nine P450s were purchased from Sigma–Aldrich Corporation (St. Louis, MO, USA), Santa Cruz Biotechnology (Dallas, TX, USA), or Cayman Chemicals (Ann Arbor, MI, USA) unless stated otherwise. The purity of all purchased compounds was higher than 97.0%. HPLC-grade acetonitrile and methanol were obtained from Burdick & Jackson Company (Morristown, NJ, USA). Caco-2 cells were supplied by the Korean Cell Line Bank (Seoul, Korea) and cultured according to the supplier’s recommendations. Transwell (24-well, 6.5 mm polycarbonate inserts, 0.4-μm pore) and cell culture reagents were purchased from Corning Life Sciences. Heparinized human plasma was obtained from donors at the Severance Hospital of Yonsei University Health System (Seoul, Korea) and stored at −80 °C prior to use.

### 2.2. Reversible Inhibition of (‒)-Sophoranone towards the Nine CYP Isoforms in Human Liver Microsomes

The inhibitory effects of SPN on CYP1A2, CYP2A6, CYP2B6, CYP2C8, CYP2C9, CYP2C19, CYP2D6, CYP2E1, and CYP3A4 were evaluated in pooled human liver microsomes through the use of specific CYP probe substrates (cocktail assay), as previously described [17,18] with a slight modification. Concentrations of each CYP probe in Table 1 were used close to their reported K_m_ values [17,18].

Briefly, a 90-µL incubation mixture, including pooled human liver microsomes (final concentration 0.1 mg/mL), 50 mM phosphate buffer (pH 7.4), each CYP-probe substrate cocktail set, and SPN (0–50 μM), was pre-incubated for 5 min at 37 °C. SPN was dissolved in methanol and spiked into the incubation mixture to a final concentration of 0.5% methanol. All P450-selective substrates (except coumarin due to solubility) were dissolved in methanol and serially diluted with methanol to the required concentrations, and the organic solvent was subsequently evaporated under a gentle stream of N_2_ gas to minimize the effects of organic solvents on CYP activities. On the other hand, coumarin dissolved in 50 mM phosphate buffer (pH 7.4) was directly added into the mixed tube. The reaction was initiated by adding 10-µL aliquot of NADPH-generating system (1.3 mM NADP^+^, 3.3 mM glucose 6-phosphate, 3.3 mM MgCl_2_, and 0.4 unit/mL glucose-6-phosphate dehydrogenase) before 15 min incubation at 37 °C in a shaking water bath. After incubation, the reactions were stopped by adding 200 µL of ice-cold acetonitrile containing 2 µM chlorpropamide as an internal standard. The incubation mixtures were centrifuged (16,000× *g*, 15 min) and 5 μL of the supernatant was injected into the LC-MS/MS system. All incubations were performed in triplicate, and the data are shown as the mean ± standard deviation. Incubation samples containing well-known CYP inhibitors for each isozyme (Table 2) in parallel were included to compare inhibitory effects, all of which appear on the US FDA list of recommended or accepted in vitro inhibitors [12,19,20,21].

Additionally, to determine whether the inhibition of CYP2C9 by SPN was substrate specific, we also examined SPN’s inhibitory effects on other CYP2C9-specific biotransformation pathways (i.e., diclofenac 4′-hydroxylation and losartan oxidation) in human liver microsomes [22,23]. Diclofenac and losartan were used at 5 μM, respectively, and other procedures were similar to those of cocktail assays.

### 2.3. Determination of the K_i_ of (‒)-Sophoranone on CYP2C9 Activity in Human Liver Microsomes

Among the nine tested CYP enzymes, SPN showed the lowest IC_50_ value for CYP2C9 (Table 2). Based on the IC_50_ values, the *K*_i_ values of SPN on CYP2C9 activity were determined. Briefly, *K*_i_ values were obtained by incubating various concentrations of two CYP2C9 probe substrates (50, 100, and 150 µM tolbutamide; or 2, 5, and 10 µM diclofenac) in the presence of 0−5 µM SPN or 0−2 µM sulfaphenazole, a well-known typical CYP2C9 inhibitor. Other procedures were similar to those of the reversible inhibition studies. All incubations were performed in triplicate, and the data are shown as the mean ± standard deviation.

### 2.4. Time-Dependent Inactivation of (‒)-Sophoranone toward the Nine CYP Isoforms in Human Liver Microsomes

Pooled human liver microsomes (1 mg/mL) were incubated with SPN (0−50 µM) for 30 min at 37 °C in the absence or presence of an NADPH-generating system (i.e., the “inactivation incubation”). After inactivation incubation, aliquots (10 μL) were transferred into fresh incubation tubes (final volume 100 μL) containing an NADPH-generating system and each P450-selective substrate cocktail set. The reaction mixtures were incubated for 15 min at 37 °C in a shaking water bath. After incubation, the reactions were stopped by adding 200 µL of ice-cold acetonitrile containing 2 µM chlorpropamide, as an internal standard. The incubation mixtures were centrifuged (16,000× *g*, 15 min) and 5 μL of the supernatant was injected into the LC-MS/MS system. All incubations were performed in triplicate, and the data are shown as the mean ± standard deviation.

### 2.5. Caco-2 Cell Permeability of (‒)-Sophoranone

Caco-2 cell permeability was assessed to predict the oral absorption of SPN. Cell culture and transport studies were performed as previously described [24,25]. Briefly, for the bi-directional transport studies, the cells were seeded at a density of 1 × 10^5^ cells/well, and the cell medium was replaced until they formed confluent monolayers. On the 25th day, the cell monolayers were washed with pre-warmed HBSS buffer. The bi-directional permeability assay was instigated by adding 10 μM for propranolol, or 10 μM and 50 μM for SPN in HBSS to an apical well (200 μL) for apical (A) to basolateral (B) transport or to a basolateral insert (800 μL) for the B to A transport. Before the experiment, the integrity of the cell monolayers was evaluated by measuring the transepithelial electrical resistance using a Millicell ohmmeter. After 2 h incubation at 37 °C, samples were withdrawn from both sides, respectively. All samples were stored at −80 °C until LC-MS/MS analysis, and all experiments were performed in triplicate.

The apparent permeability coefficient (P_app_) was calculated using the following equation.

P_app_ = (V_r_/C_0_) × (1/A) × ([Drug]/t)

where, V_r_ is the volume of medium in the receiver chamber, C_0_ is the donor compartment concentration at time zero, A is the area of the cell monolayer, t is the treatment time of the drug, and [Drug] is the drug concentration in the receiver chamber.

### 2.6. Effects of (‒)-Sophoranone on the Pharmacokinetics of Diclofenac in Rats

In this study, we investigated whether SPN, an in vitro potent inhibitor of CYP2C9, affects the pharmacokinetics of diclofenac in rats. Male Sprague–Dawley rats (8 weeks, 270–290 g) were purchased from Orient Bio (Sungnam, Gyeonggi-do, Korea), and the protocol for pharmacokinetic interaction studies in rats was approved by the Institutional Animal Care and Use Committee (IACUC-CUK) at The Catholic University of Korea (Approval No. 2019-021, approved 31 May 2019). The procedures used for housing and handling were previously reported [18]. Before administration, rats were fasted for 12 h with free access to water. The carotid arteries of each rat were cannulated with a polyethylene tube (Clay Adams, Franklin Lakes, NJ, USA) for blood sampling. Each rat was individually housed in a rat metabolic cage and allowed to recover from anesthesia for 4–5 h prior to the start of the experiment. The rats were divided into two groups: (1) diclofenac alone (*n* = 6) and (2) SPN and diclofenac co-administration (*n* = 6). SPN was suspended in dimethylsulfoxide:PEG400:distilled water (5:60:35, *v*/*v*/*v*) and administered by oral gavage at a dose of 75 mg/kg in a volume of 5 mL/kg. Fifteen minutes after oral administration of SPN, 2 mg/kg diclofenac was dissolved in normal saline and administered by oral gavage. Approximately 0.25 mL of blood from each rat was collected into an Eppendorf tube before diclofenac dosing (0 min), and at 3, 5, 10, 15, 30, 45, 60, 90, 120, 180, 240, 360, and 480 min post-dosing. The blood samples were immediately centrifuged at 13,000× *g* for 5 min at 4 °C. The plasma samples were divided into two Eppendorf tubes by 50 μL and stored at −80 °C until LC-MS/MS analysis. After the experiments, the rats were euthanized with CO_2_.

### 2.7. Determination of the Unbound Fraction of (‒)-Sophoranone in Plasma and Human Liver Microsomes

The plasma or liver microsomal protein bindings were performed using a rapid equilibrium dialysis device and cellulose membranes with a molecular weight cutoff of 8000 (Thermo Scientific, Rockford, IL, USA) [17]. The rat and human plasma samples (200 μL) containing SPN at 10 and 50 μM, respectively, were dialyzed against a dialysis buffer, phosphate-buffered saline (PBS, 400 μL). The loaded dialysis plate was covered with sealing tape, placed on an orbital shaker at approximately 200 rpm, and incubated at 37 °C for 4 h. Thereafter, samples (100 μL) from both PBS and plasma chambers were collected and mixed with an equal volume of blank plasma and PBS, respectively. All samples were stored at −80 °C until LC-MS/MS analysis. The unbound fraction of SPN in human (or rat) plasma was calculated by dividing the SPN concentration in PBS by that in plasma.

The human liver microsomal incubation mixtures (final concentration 0.1 mg/mL) without NADPH generating system were used to determine the unbound fraction of SPN. Other procedures were similar to those of plasma protein binding assay.

### 2.8. LC-MS/MS Analysis

#### 2.8.1. In Vitro Samples

Metabolites of nine P450-selective substrates were analyzed using a Shimadzu Nexera X2 UPLC system coupled to an LCMS-8050 triple quadruple mass spectrometer (Shimadzu Corporation, Kyoto, Japan) equipped with an electrospray ionization interface as previously described with a slight modification [17,18]. Separation was performed on a reversed-phase column (Luna C_18_, 50 mm × 2.0 mm i.d.; 3 μm particle size; Phenomenex, Torrance, CA, USA) maintained at 40 °C. The mobile phase consisted of distilled water containing 0.1% formic acid (A) and acetonitrile containing 0.1% formic acid (B), with a flow rate of 0.5 mL/min. The gradient elution program used was as follows: (1) Mobile phase A was set to 95% at 0 min, (2) a linear gradient was run to 5% in 2.6 min, and (3) a linear gradient was run to 95% in 3.0 min and re-equilibrated for 2 min. The total run time was 5 min. The optimized compound-dependent parameters of the metabolites of the nine P450-selective substrates and the internal standard are listed in Table 1. Three-day validations were performed to confirm the effectiveness of the LC-MS/MS system for simultaneous determination of the nine P450-selective substrate metabolites at the respective ranges of 0.01–10 μM in blank microsomal incubation mixtures. We found that the precision (≤12.1%) and accuracy (95.4–110.2%) values were within acceptable ranges. Supplemental Figure S1 shows the representative LC-MS/MS chromatograms of a human liver microsomal incubation sample containing nine P450-selective metabolites and an internal standard.

The auto-optimized mass transitions were *m*/*z* 312 > 231 and *m*/*z* 437 > 207.1 for quantification of 4′-hydroxydiclofenac and losartan carboxylic acid, respectively. HPLC conditions were the same as those in the cocktail assay.

#### 2.8.2. In Vivo Samples

The plasma concentrations of diclofenac and 4′-hydroxydiclofenac were determined by a previously reported LC-MS/MS method [26] with some modifications. Briefly, 50 μL aliquots of plasma were extracted with 300 μL aliquots of acetonitrile containing chlorpropamide (internal standard), followed by LC-MS/MS (Shimadzu Corporation). Chromatographic separation was performed on a Phenomenex Luna C_18_ column (100 × 2.00 mm; 3.0 μm). The isocratic mobile phase consisted of 0.1% formic acid in distilled water (A) and 0.1% formic acid in acetonitrile (B) (45:55, *v*/*v*), with a flow rate of 0.3 mL/min. The transitions were *m*/*z* 296.0 > 214.0 for diclofenac, *m*/*z* 312 > 231 for 4′-hydroxydiclofenac, and *m*/*z* 277 > 111 for the internal standard. The data acquisition was computed using LabSolutions LCMS Ver.5.6 (Shimadzu Corporation). The calibration curves for diclofenac and 4′-hydroxydiclofenac were linear (*r* ≥ 0.996) over the concentration range of 20–5000 ng/mL.

The LC-MS/MS condition for the determination of SPN in plasma was the same with a previously reported method [27]. The calibration curve for SPN was linear (*r* ≥ 0.995) over the concentration range of 1–250 ng/mL.

### 2.9. Analysis of Inhibition Kinetics and Pharmacokinetic Parameters

The IC_50_ values were calculated via nonlinear least-squares regression analysis from logarithmic plots of inhibitor concentration versus percentage of activity remaining after inhibition, using SigmaPlot (ver. 14.0; Systat Software Inc, Chicago, IL, USA). The *K*_i_ values were determined from the equations for a single substrate single inhibitor model and the software available in the SigmaPlot Enzyme Kinetics module. Competitive, non-competitive, uncompetitive, or mixed inhibition models were evaluated and ranked according to the best fit based on Akaike Information Criterion (AIC) values. For visual inspection, the data were presented as Dixon plots.

Pharmacokinetic parameters were calculated by a non-compartmental analysis using WinNonlin Professional software (version 5.2, Pharsight Corp., Mountain View, CA, USA) that used the total area under the plasma concentration–time curve from time zero to infinity (AUC_∞_) or the last measured time (AUC_t_). The logarithmic trapezoidal rule was used during the declining plasma level phase and the linear trapezoidal rule was used for the rising plasma-level phase. The peak plasma concentration (C_max_) and time to reach C_max_ (T_max_) were read directly from the experimental data. Statistically significant differences were recognized at *p* < 0.05.

## 3. Results

### 3.1. Reversible Inhibition of (‒)-Sophoranone toward the Nine CYP Isoforms in Human Liver Microsomes

**Figure 2:**
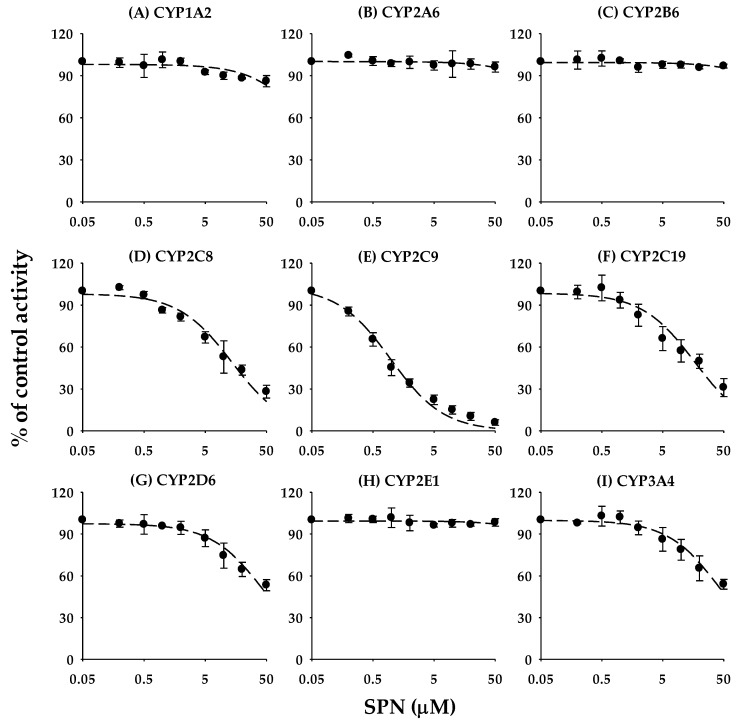
Inhibition curves of SPN on the nine major P450 activities in human liver microsomes using substrate cocktails including CYP1A2 for phenacetin *O*-deethylase (**A**), CYP2A6 for coumarin 7-hydroxylase (**B**), CYP2B6 for bupropion hydroxylase (**C**), CYP2C8 for rosiglitazone *p*-hydroxylase (**D**), CYP2C9 for tolbutamide 4-hydroxylase (**E**), CYP2C19 for omeprazole 5-hydroxylase (**F**), CYP2D6 for dextromethorphan *O*-demethylase (**G**), CYP2E1 for chlorzoxazone 6-hydroxylase (**H**), and CYP3A4 for midazolam 1′-hydroxylase (**I**). The activity is expressed as a percentage of remaining activity compared with the control, no containing SPN. Data are the mean ± standard deviation of triplicate incubations. The dashed lines represent the best fit to the data with non-linear regression.

The inhibitory effects of SPN on the activities of nine CYP isozymes (CYP1A2, CYP2A6, CYP2B6, CYP2C8, CYP2C9, CYP2C19, CYP2D6, CYP2E1, and CYP3A4) in human liver microsomes are shown in Figure 2, and the IC_50_ values are listed in Table 2. The IC_50_ values for the positive controls used in the reversible inhibition studies were in an acceptable degree of accuracy with published values [12,19,20,21]. Of the P450 isoforms tested, SPN exerted the strongest inhibitory effect on CYP2C9-catalyzed tolbutamide hydroxylation, with an IC_50_ value of 0.966 ± 0.149 μM (Table 2). SPN showed weak inhibitory effects toward CYP2C8 and CYP2C19, with IC50 values of 13.6 ± 3.15 μM and 16.8 ± 3.21 μM, respectively. However, SPN had no apparent inhibitory effects toward the other CYPs tested (Table 2); the residual enzyme activities at the highest tested concentration (50 μM) were greater than 80%, except for CYP2D6 (53.9 ± 3.53%) and CYP3A4 (53.3 ± 4.00%) (Figure 2).

**Figure 3:**
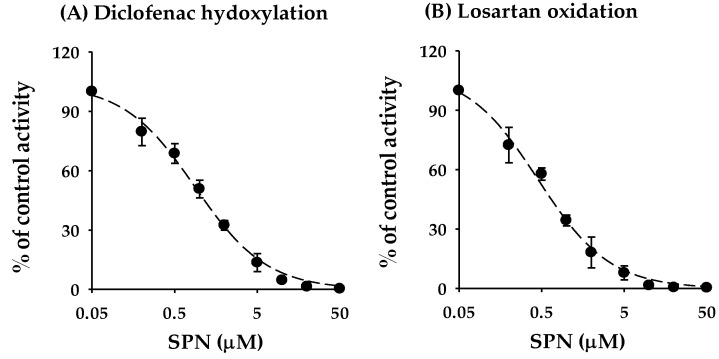
Inhibition curves of SPN on the CYP2C9-catalyzed diclofenac 4′-hydroxylation (**A**) and losartan oxidation (**B**) activities in human liver microsomes. Data are the mean ± standard deviation of triplicate incubations. The dashed lines represent the best fit to the data with non-linear regression.

To determine whether the inhibitory effects of SPN on CYP2C9 was substrate specific, we examined the inhibitory effects on other CYP2C9-specific biotransformation pathways (i.e., diclofenac 4′-hydroxylation and losartan oxidation) and found that SPN also markedly inhibited their activities, with IC_50_ values of 0.879 ± 0.0888 μM and 0.455 ± 0.0486 μM, respectively, (Figure 3).

### 3.2. Determination of the K_i_ of (‒)-Sophoranone for CYP2C9 Activity

**Figure 4:**
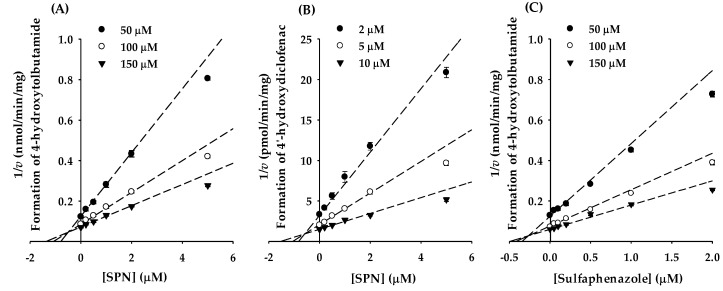
Dixon plots to determine *K*_i_ values of SPN on the CYP2C9 enzyme activity, using tolbutamide (**A**) or diclofenac (**B**) as substrates. The well-known inhibitor of CYP2C9, sulfaphenazole, is used as a positive control (**C**) using tolbutamide as a substrate. The concentrations of tolbutamide were determined 50 (●), 100 (○), and 150 (▼) μM, respectively; diclofenac was used at 2 (●), 5 (○), and 10 (▼) μM, respectively. *v* represents formation rate of 4-hydroxytolbutamide (nmol/min/mg protein) or 4′-hydroxydiclofenac (pmol/min/mg protein). Data are the mean ± standard deviation of triplicate incubations. The dashed lines of SPN (**A**,**B**) and sulfaphenazole (**C**) all fit well to competitive inhibition types.

Based on the lowest IC_50_ value for CYP2C9, to characterize the type of reversible inhibition of CYP2C9 by SPN, enzyme kinetic experiments were performed in the presence of various concentrations of SPN and tolbutamide, or diclofenac. Otherwise, identical samples containing a known potent CYP2C9 inhibitor (sulfaphenazole), were included in the analysis. Representative Dixon plots of CYP2C9 inhibition by SPN and sulfaphenazole in human liver microsomes are shown in Figure 4, and the Ki values are summarized in Supplemental Table S1. Using a nonlinear regression analysis, SPN demonstrated competitive inhibition against CYP2C9-catalyzed tolbutamide hydroxylation or diclofenac hydroxylation, with calculated *K*_i_ values of 0.503 ± 0.0383 μM and 0.587 ± 0.0470 μM (Figure 4A,B). Sulphafenazole competitively inhibited CYP2C9 with a *K*_i_ value of 0.267 ± 0.0170 μM (Figure 4C), which was similar to a previously reported value [28].

### 3.3. Time-Dependent Inactivation of (‒)-Sophoranone towards the Nine CYP Isoforms in Human Liver Microsomes

**Figure 5:**
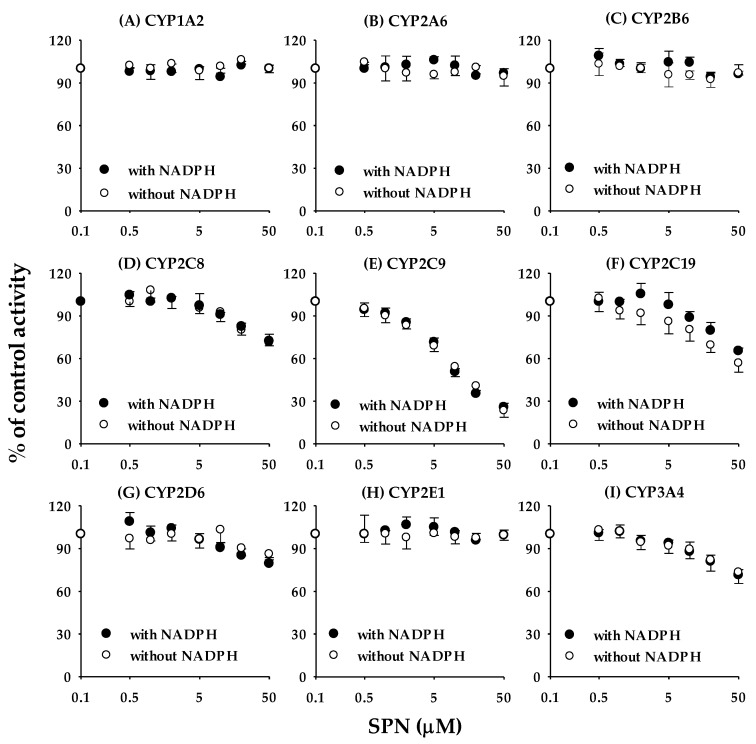
Time-dependent inhibition curves of SPN on the nine major P450 activities in human liver microsomes using substrate cocktails after 30 min pre-incubation with the presence (●) or absence (○) of an NADPH-generating system. Data are the mean ± standard deviation of triplicate incubations.

The IC_50_ shift method incorporating a dilution is one of the most efficient and convenient methods for evaluating time-dependent inhibitory effects. A shift in IC_50_ to a lower value (“shift”) with pre-incubation indicates time-dependent inactivation [29,30,31]. After 30 min pre-incubation of SPN with human liver microsomes in the presence of NADPH, no obvious shift in IC_50_ was observed for inhibition of the nine CYPs (Figure 5), suggesting that SPN is not a time-dependent inactivator for the nine CYPs.

### 3.4. Caco-2 Cell Permeability of (‒)-Sophoranone

A bi-directional permeability assay using Caco-2 monolayer cells was performed to predict the intestinal absorption of SPN. SPN showed very low permeability in both directions (from A-to-B and B-to-A). The calculated P_app_ values of SPN from A-to-B were (0.115 ± 0.0369) × 10^−6^ cm/s and (0.172 ± 0.0488) × 10^–6^ cm/s at 10 μM and 50 μM, respectively, (*n* = 3, each). These results indicated that SPN is poorly absorbed in vivo. The P_app_ values from B-to-A were (0.101 ± 0.00444) × 10^−6^ cm/s at 10 μM (*n* = 3) and (0.152 ± 0.0353) × 10^−6^ cm/s at 50 μM (*n* = 3). SPN was not a substrate for efflux transporters, that is, P-gp and BCRP, as the efflux ratio (B-to-A/A-to-B) is less than 2. The P_app_ of propranolol, a reference high permeable compound, from A-to-B and B-to-A were (26.8 ± 3.31) × 10^−6^ cm/s and (21.5 ± 2.19) × 10^−6^ cm/s, respectively, (*n* = 3, each), similar to the reported values [24,25].

### 3.5. Effects of (‒)-Sophoranone on the Pharmacokinetics of Diclofenac in Rats

We conducted pharmacokinetic studies to investigate the effects of SPN on the pharmacokinetics of diclofenac in rats. Findings in the literature on the dried *S. tonkinensis* herbs indicate that a recommended daily dose for an adult human with the body weight of 60 kg were to be 6–10 g [32], which correlated to the equivalent dose ranges in rats, 0.620–1.03 g/kg [33]. He et al. [2] reported that the average contents of SPN in various *S. tonkinensis* samples were found to be approximately 2.53 mg/g (0.0253%). Reflecting this content, the dosage in rats, 0.620–1.03 g/kg of the dried herb, might be consistent with 15.7–26.1 mg/kg in terms of SPN. Thus, in this study, the SPN dose of 75 mg/kg was used in rats, which is approximately 2.87- to 4.87-fold greater than the recommended human dose.

**Figure 6:**
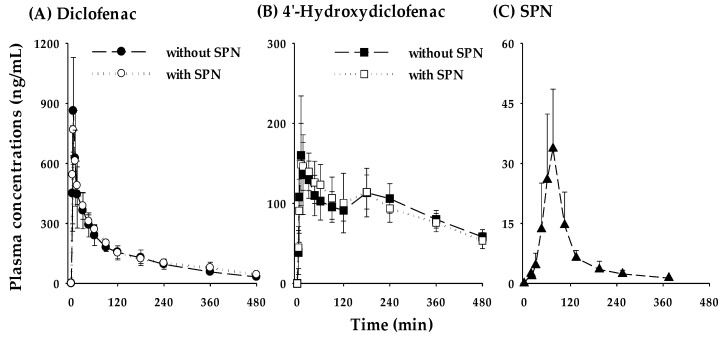
Mean plasma concentrations of diclofenac (**A**) and 4′-hydroxydiclofenac (**B**) after oral administration of diclofenac at a dose of 2 mg/kg without (●, *n* = 6) or with (○, *n* = 6) oral dosing of SPN (75 mg/kg) to rats. Mean plasma concentrations of SPN (**C**) after co-administration of SPN (75 mg/kg) and diclofenac (2 mg/kg) to rats (▲, *n* = 6). Vertical bars mean standard deviation.

The mean plasma concentration-time profiles of diclofenac and 4-hydroxydiclofenac after oral administration of diclofenac (2 mg/kg) in the absence or presence of oral co-administration of SPN (75 mg/kg) in rats are illustrated in Figure 6, and the relevant pharmacokinetic parameters are shown in Table 3. The plasma levels of diclofenac and 4-hydroxydiclofenac were similar in both groups (Figure 6A,B). Likewise, no significant differences were observed in any other pharmacokinetic parameter of diclofenac and 4′-hydroxydiclofenac (Table 3). The in vivo marker for CYP2C9 activity, expressed as the molar AUC ratio of 4′-hydroxydiclofenac to diclofenac, was not significant (0.799 ± 0.167 versus 0.904 ± 0.0534; *p* value of 0.215) in the presence or absence of SPN (Table 3). In the treatment group with co-administration of SPN, the C_max_ of SPN was found to be 33.7 ± 14.8 ng/mL (0.0732 ± 0.0321 μM) at approximately 60–75 min post-dose (Figure 6C). Given the *K*_i_ values of SPN on CYP2C9 activity (0.503 ± 0.0383 μM for tolbutamide hydroxylation and 0.587 ± 0.0470 μM for diclofenac hydroxylation), the plasma concentrations of SPN are too low to inhibit CYP2C9-mediated metabolism of diclofenac in vivo. Overall, the co-administration of SPN did not alter the pharmacokinetics of diclofenac and 4′-hydroxydiclofenac.

### 3.6. Determination of the Unbound Fraction of (‒)-Sophoranone in Plasma and Human Liver Microsomes

SPN was extensively bound to plasma proteins, regardless of species. The free fractions (%) of SPN at 10 and 50 μM in human plasma were 0.0457 ± 0.00612% and 0.0927 ± 0.0400%, respectively, (*n* = 3, each). Similarly, when 10 and 50 μM SPN were added to the rat plasma, the free fractions were 0.0380 ± 0.0102% and 0.0531 ± 0.0149%, respectively, (*n* = 3, each). After adding 10 and 50 μM SPN to rat and human plasma, free fractions remained relatively unchanged, suggesting that SPN has no binding saturation in plasma.

SPN also exhibited marked non-specific bindings to human liver microsomes, although to a lesser extent than those in human plasma. The unbound fractions of SPN at 10 and 50 μM were calculated to be 0.621 ± 0.0405% and 0.724 ± 0.170%, respectively (*n* = 3, each), at a microsomal protein concentration of 0.1 mg/mL.

## 4. Discussion

This study focused on the in vitro and in vivo inhibitory effects of SPN on human CYPs, especially CYP2C9. We screened the inhibitory effects of SPN on the major human CYP isoforms (CYP1A2, CYP2A6, CYP2B6, CYP2C8, CYP2C9, CYP2C19, CYP2D6, CYP2E1, and CYP3A4) in human liver microsomes. Of the nine tested CYP isoforms, SPN exerted the strongest inhibitory effect on CYP2C9 activity, with the lowest IC_50_ value of 0.966 ± 0.149 μM (Table 2; Figure 2). In addition to CYP2C9, SPN mildly inhibited several CYP enzymes, with potency ranked in the order CYP2C8 > CYP2C19; the IC_50_ values were 13.6 ± 3.15 μM and 16.8 ± 3.21 μM, respectively (Table 2; Figure 2). Although the IC_50_ values could not been calculated, SPN also appears to weakly inhibit CYP2D6 and CYP3A4; the residual enzyme activities at the highest tested concentration (50 μM) were 53.9 ± 3.53% and 53.3 ± 4.00%, respectively (Figure 2). No apparent inhibition of the other CYPs (CYP1A2, CYP2A6, CYP2B6, and CYP2E1) was observed (Figure 2). SPN also strongly inhibited other CYP2C9-catalyzed diclofenac 4′-hydroxylation and losartan oxidation activities (Figure 3). The inhibition mechanisms of SPN on CYP2C9-catalyzed tolbutamide 4-hydroxylation and diclofenac 4′-hydroxylation activities were both competitive, with *K*_i_ values of 0.503 ± 0.0383 μM and 0.587 ± 0.0470 μM, respectively. Pre-incubation of SPN for 30 min with human liver microsomes and an NADPH-generating system did not alter the inhibition potencies against the nine CYPs, suggesting that SPN is not a time-dependent inactivator.

The reversible inhibition of SPN-mediated CYP3A4 activity was less consistent with the published literature. Li et al. [16] reported that among 44 tested flavonoids, SPN inhibited CYP3A4-catalyzed bufalin 5′-hydroxylation activity with a *K*_i_ value of 2.17 ± 0.29 μM. They only focused on the in vitro inhibitory potentials of several flavonoids against CYP3A4 activity. To the best of our knowledge, to date, bufalin has not been used as the in vitro probe substrate for the CYP3A4 activity, and the reference material of 5′-hydroxybufalin is not commercially available. Because of the presence of several binding regions within the CYP3A4 active site, multiple probe substrates are often used for in vitro CYP3A4-mediated drug–drug interaction studies, including midazolam, nifedipine, and testosterone [34]. In that study, when other CYP3A4 substrates were tested, the ranges of IC_50_ values by SPN were reported to be 5.62–38.4 μM [16]. Additionally, we examined the inhibitory effect on another CYP3A4-catalyzed testosterone 6β-hydroxylation and found that SPN also inhibited the activity with an IC_50_ value of 31.5 ± 4.79 μM, which showed a higher percentage inhibition compared to midazolam (data not shown). Altogether, the in vitro CYP3A4 inhibition by SPN seemed to be substrate-specific.

Generally, alterations in the activities of hepatic CYPs through in vitro inhibition or induction represent the major mechanisms underlying pharmacokinetic drug–drug interactions [11,12,13]. It has been estimated that CYP2C9 is responsible for the metabolic clearance of up to 15–20% of all drugs undergoing phase I metabolism, including clinically important drugs such as *S*-warfarin, phenytoin, tolbutamide, losartan, and several anti-inflammatory drugs [23,35]. Considering that SPN is a potent CYP2C9 inhibitor in vitro, there may be potential for herb–drug interactions between SPN and CYP2C9 substrates after concomitant oral administration.

Using the in vitro reversible inhibition results, a clinical drug–drug interaction risk was initially predicted by the basic static model approach, as recommended by the EMA [36] and US FDA [37] with calculating the R_1_ value (R_1_ = 1 + [I_max,u_/*K*_i,u_]), which representing the predicted AUC ratio in the presence or absence of inhibitor. Where, I_max,u_ (C_max,u_) is maximal free plasma concentration of the inhibitor and *K*_i,u_ is the unbound in vitro inhibition constant. However, little information is yet to be reported on the C_max_ values of SPN after oral administration of SPN. As stated in the Introduction, from our previous study, the C_max_ of SPN was reported to be 13.1 ng/mL in rats after oral dosing of 12.9 mg/kg SPN in rats [8]. Thus, we investigated whether SPN affects the pharmacokinetics of diclofenac and 4′-hydroxydiclofenac, produced by hepatic CYP2C9 enzyme, in rats. In the group that received co-administration of SPN (75 mg/kg), the C_max_ of SPN was found to be 33.7 ± 14.8 ng/mL (0.0732 ± 0.0321 μM) at 60–75 min (Figure 6C). These results suggest that SPN has low oral bioavailability. The calculated values of I_max, u_ and *K*_i,u_ for SPN used in this study were 0.0420 ± 0.0184 nM and 3.39 ± 0.258 nM (3.95 ± 0.316 nM for diclofenac 4′-hydroxylation), respectively. Considering these values, the R_1_ value of SPN for the inhibition of CYP2C9 in vitro was calculated as 1.0124 (*K*_i_, u for tolbutamide 4-hydroxylation) or 1.0106 (K_i, u_ for diclofenac 4′-hydroxylation) which are both below the EMA and US FDA cut-off criteria of R_1_, 1.02 [36,37], indicating that the potential for clinically relevant drug interaction-mediated CYP2C9 inhibition by SPN may be low and no clinical interaction studies are warranted. In our results, also no significant differences were observed in any of the other pharmacokinetic parameters of diclofenac and 4′-hydroxydiclofenac in rats in the absence or presence of oral co-administration of SPN at a dose of 75 mg/kg (Table 3). Furthermore, the molar metabolic conversion ratio, expressed as AUC_4′-hydroxydiclofenac_/AUC_diclofenac_, which indicated a causal factor for the evaluation of the capacities of CYP2C9 activity in vivo, did not show significant differences (0.799 ± 0.167 versus 0.904 ± 0.0534) in both groups (Table 3).

To explain the lack of in vitro–in vivo correlation, we assessed two factors that could limit the accuracy of in vitro models in predicting metabolic drug interactions in vivo, which were SPN’s degree of plasma protein binding and its permeability in Caco-2 cells. We found that SPN was extensively bound in both human and rat plasma proteins (>99.9%) with a mean unbound fraction value of 0.0574% in the range of 10 and 50 μM. Thus, taking the plasma protein binding of SPN into account, the unbound maximum concentrations of SPN in plasma might be 0.0420 ± 0.0184 nM, which is much lower than the unbound *K*_i_ values of SPN in vitro. Some drugs that indicate in vitro–in vivo discrepancy because of high plasma protein bindings have been reported [38,39,40]. Tolfenamic acid strongly inhibited CYP1A2 in vitro but not in vivo because of high plasma protein binding (99.7%) [38]. Montelukast is a very potent inhibitor of CYP2C8 in vitro with *K*_i_ values ranging from 0.0092–0.15 μM [41]. However, in humans, montelukast has had no effect on the pharmacokinetics of the CYP2C8 substrates, pioglitazone [39] and rosiglitazone [40]. The high degree of protein binding of montelukast in plasma (>99.7%) is similar to that of tolfenamic acid and explicitly explains the lack of its in vivo interaction, irrespective of its strong inhibitor potency in vitro. The Caco-2 cell model is widely used to predict the absorption across the intestinal barrier, and a good correlation between its oral absorption in humans and its apparent permeability (P_app_) across the Caco-2 cell barrier has been shown [24,25]. A recent study has provided some updated guidelines on how permeability values might correlate with human oral absorption: Low permeability (0–20% absorbed) is correlated to P_app_ values < 1–2 × 10^−6^ cm/s; moderate permeability (20–80% absorbed) to P_app_ values < 2–10 × 10^−6^ cm/s; and high permeability (80–100% absorbed) to P_app_ values > 10 × 10^−6^ cm/s [42]. Propranolol had >90% human absorption and exhibited high permeability with a P_app_ value of (26.8 ± 3.31) × 10^−6^ cm/s in our assay. SPN exhibited a very low permeability with mean P_app_ values of 0.115 × 10^−6^ cm/s (0.429% of propranolol P_app_) and 0.172 × 10^−6^ cm/s (0.642% of propranolol P_app_) at 10 and 50 μM, respectively, indicating that it is poorly absorbed in vivo. SPN was not a substrate for efflux transporters, that is, P-gp and BCRP, as the efflux ratio (B-to-A/A-to-B) is less than 2.

Overall, SPN is a potent inhibitor of CYP2C9 in vitro but not in vivo. This apparent discrepancy is due to the extensive plasma protein binding and very low permeability of SPN, which resulted in poor oral absorption. These approaches could help in making more reliable in vitro–in vivo extrapolations about the risk of in vivo inhibition potential. In conclusion, these findings have provided useful information on the safe and effective use of SPN in clinical practice.

## Figures and Tables

**Table 1:** Optimized mass parameters for the detection of metabolites of the nine P450-probe substrates and internal standard used in the cocktail assays.

CYPs	Probe Substrates	K_m_ (μM)	Metabolite	ESI ^a^	Q1 Ion (*m/z*)	Q3 Ion (*m/z*)	Q1 Pre-bias (V)	CE ^b^ (eV)	Q3 Pre-bias (V)
1A2	Phenacetin	50	Acetaminophen	+	152	110.2	−14	−12	−19
2A6	Coumarin	5	7-Hydroxycoumarin	+	163	107	−15	−35	−15
2B6	Bupropion	50	6-Hydroxybupropion	+	256	238	−15	−35	−15
2C8	Rosiglitazone	10	*p*-Hydroxyrosiglitaonze	+	374	151	−15	−35	−15
2C9	Tolbutamide	100	4-Hydroxytolbutamide	+	287	87	−15	−35	−15
2C19	Omeprazole	20	5-Hydroxyomeprazole	+	362	214	−13	−13	−22
2D6	Dextromethorphan	5	Dextrorphan	+	258	157	−15	−35	−15
2E1	Chlorzoxazone	50	6-Hydroxychlorzoxazone	−	184	119.9	18	15	24
3A4	Midazolam	2	1′-Hydroxymidazolam	+	342	203	−15	−35	−15
Chlorpropamide (Internal standard)		2		+	277	111	−15	−20	−15
				−	275	190	15	35	15

The optimized ion spray voltage was 4 kV and a nebulizing gas flow of 3 L/min, heating gas flow of 10 L/min, an interface temperature of 300 °C, desolvation line temperature of 250 °C, heating block temperature of 400 °C, and a drying gas flow rate of 10 L/min. ^a^ ESI, electrospray ionization mode; ^b^ CE, collision energy.

**Table 2:** IC_50_ values of well-known CYP inhibitors and SPN in reversible inhibition studies using a cocktail assay (*n* = 3).

CYPs	IC_50_ Values (μM)		
	Well-Known Inhibitors		SPN
1A2	α-Naphthoflavone	0.0458 ± 0.00694	>50 ^a^
2A6	Tryptamine	2.98 ± 0.635	>50 ^a^
2B6	Ticlopidine	2.19 ± 0.513	>50 ^a^
2C8	Quercetin	8.51 ± 0.958	13.6 ± 3.15
2C9	Sulfaphenazole	0.677 ± 0.109	0.966 ± 0.149
2C19	*S*-benzylnirvanol	0.215 ± 0.0228	16.8 ± 3.21
2D6	Quinidine	0.127 ± 0.0192	>50
2E1	Diethyldithiocarbamate	12.0 ± 3.67	>50 ^a^
3A4	Ketoconazole	0.0404 ± 0.00821	>50

Data represent the mean ± standard deviation of triplicate. ^a^ The remaining activities at the highest concentration tested, 50 μM, were greater than 80%.

**Table 3:** Mean (± standard deviations) pharmacokinetic parameters of diclofenac and 4′-hydroxydiclofenac after oral administration of diclofenac at a dose of 2 mg/kg without or with oral administration of SPN (75 mg/kg) to rats.

Parameters	Without SPN (*n* = 6)	With SPN (*n* = 6)
Diclofenac		
AUC_t_ (μg min/mL) ^a^	63.8 ± 6.28	69.4 ± 2.98
AUC_∞_ (μg min/mL) ^b^	71.7 ± 9.16	80.8 ± 7.78
t_1/2_ (min) ^c^	153 ± 60.1	173 ± 41.5
C_max_ (ng/mL) ^d^	882 ± 245	787 ± 104
T_max_ (min) ^e^	5 (3–5)	5 (3–5)
4′-hydroxydiclofenac		
AUC_t_ (μg min/mL)	44.8 ± 6.38	44.5 ± 7.24
AUC_∞_ (μg min/mL)	68.6 ± 12.1	67.6 ± 12.9
t_1/2_ (min)	278 ± 70.0	296 ± 59.7
C_max_ (ng/mL)	180 ± 55.2	173 ± 40.8
T_max_ (min)	10 (10–30)	15 (10–30)
Metabolic conversion ratio ^f^		
AUC_∞, 4′-hydroxydiclofenac_/AUC_∞, diclofenac_	0.904 ± 0.0534	0.799 ± 0.167

No significant differences were observed in all pharmacokinetic parameters of diclofenac and 4′-hydroxydiclofenac. ^a^ Total area under the plasma concentration–time curve from time zero to time last sampling time; ^b^ total area under the plasma concentration–time curve from time zero to infinity; ^c^ terminal half-life; ^d^ peak plasma concentration; ^e^ time to reach C_max_. Median (ranges); ^f^ the metabolic conversion ratio, AUC_∞,4′-hydroxydiclofenac_/AUC_∞,diclofenac_, was calculated based on a molar basis.

## Supplementary Materials

The following is available online at https://www.mdpi.com/1999-4923/12/4/328/s1, Figure S1: Typical LC-MS/MS chromatograms for the formed metabolites of the nine CYP-specific probe in human liver microsomes and their internal standard in the positive electrospray ionization mode. Table S1: *K*_i_ values and inhibition types for CYP2C9 by SPN and sulfaphenazole in human liver microsomes (*n* = 3).

## Previous Presentation of Information

A portion of the information in this manuscript has been presented as a poster in a conference previously: Soo Hyun Jang, Yu Fen Zheng, Chae Bin Lee, Soo Kyung Bae. 2019. Cytochrome P450 2C9 inhibition by sophoranone in human liver microsomes. 15th Euro-Global Summit on Toxicology and Applied Pharmacology, Berlin, Germany.

## References

[1] Ding P.L., He C.M., Cheng Z.H., Chen D.F. (2018). Flavonoids rather than alkaloids as the diagnostic constituents to distinguish *Sophorae Flavescentis Radix* from *Sophorae Tonkinensis Radix* et Rhizoma: An HPLC fingerprint study. Chin. J. Nat. Med..

[2] He C.M., Cheng Z.H., Chen D.F. (2013). Qualitative and quantitative analysis of flavonoids in *Sophora tonkinensis* by LC/MS and HPLC. Chin. J. Nat. Med..

[3] Yoo H., Kang M., Pyo S., Chae H.S., Ryu K.H., Kim J., Chin Y.W. (2017). SKI3301, a purified herbal extract from *Sophora tonkinensis*, inhibited airway inflammation and bronchospasm in allergic asthma animal models in vivo. J. Ethnopharmacol..

[4] Lee J.W., Lee J.H., Lee C., Jin Q., Lee D., Kim Y., Hong J.T., Lee M.K., Hwang B.Y. (2015). Inhibitory constituents of *Sophora tonkinensis* on nitric oxide production in RAW 264.7 macrophages. Bioorg. Med. Chem. Lett..

[5] Kajimoto S., Takanashi N., Kajimoto T., Xu M., Cao J., Masuda Y., Aiuchi T., Nakajo S., Ida Y., Nakaya K. (2002). Sophoranone, extracted from a traditional Chinese medicine Shan Dou Gen, induces apoptosis in human leukemia U937 cells via formation of reactive oxygen species and opening of mitochondrial permeability transition pores. Int. J. Cancer.

[6] Yang X., Deng S., Huang M., Wang J., Chen L., Xiong M., Yang J., Zheng S., Ma X., Zhao P. (2017). Chemical constituents from *Sophora tonkinensis* and their glucose transporter 4 translocation activities. Bioorg. Med. Chem. Lett..

[7] Atta-Ur-Rahman, Haroone M.S., Tareen R.B., Mohammed Ahmed Hassan O.M., Jan S., Abbaskhan A., Asif M., Gulzar T., Al-Majid A.M., Yousuf S. (2012). Secondary metabolites of Sophora mollis subsp. griffithii (Stocks) Ali. Phytochem. Lett..

[8] Jang S.M., Bae S.H., Choi W.K., Park J.B., Kim D., Min J.S., Yoo H., Kang M., Ryu K.H., Bae S.K. (2015). Pharmacokinetic properties of trifolirhizin, (-)-maackiain, (-)-sophoranone and 2-(2,4-dihydroxyphenyl)-5,6-methylenedioxybenzofuran after intravenous and oral administration of *Sophora tonkinensis* extract in rats. Xenobiotica.

[9] Rekić D., Reynolds K.S., Zhao P., Zhang L., Yoshida K., Sachar M., Piquette Miller M., Huang S.M., Zineh I. (2017). Clinical drug-drug interaction evaluations to inform drug use and enable drug access. J. Pharm. Sci..

[10] Bjornsson T.D., Callaghan J.T., Einolf H.J., Fischer V., Gas L., Grimm S., Kao J., King S.P., Miwa G., Ni L. (2003). The conduct of in vitro and in vivo drug-drug interaction studies: A pharmaceutical research and manufactures of America (PhRMA) perspective. Drug Metab. Dispos..

[11] Lin J.H., Lu A.Y. (1998). Inhibition and induction of cytochrome P450 and the clinical implications. Clin. Pharmacokinet..

[12] Peng Y., Wu H., Zhang X., Zhang F., Qi H., Zhong Y., Wang Y., Sang H., Wang G., Sun J. (2015). A comprehensive assay for nine major cytochrome P450 enzymes activities with 16 probe reactions on human liver microsomes by a single LC/MS/MS run to support reliable in vitro inhibitory drug-drug interaction evaluation. Xenobiotica.

[13] Wienkers L.C., Heath T.G. (2005). Predicting in vivo drug interactions from in vitro drug discovery data. Nat. Rev. Drug Discov..

[14] Chen Q., Zhang T., Wang J.F., Wei D.Q. (2011). Advances in human cytochrome P450 and personalized medicine. Curr. Drug Metab..

[15] Cai J., Ma J., Xu K., Gao G., Xiang Y., Lin C. (2015). Effect of Radix Sophorae Tonkinensis on the activity of cytochrome P450 isoforms in rats. Int. J. Clin. Exp. Med..

[16] Li Y., Ning J., Wang Y., Wang C., Sun C., Huo X., Yu Z., Feng L., Zhang B., Tian X. (2018). Drug interaction study of flavonoids toward CYP3A4 and their quantitative structure activity relationship (QSAR) analysis for predicting potential effects. Toxicol. Lett..

[17] Cho D.Y., Bae S.H., Lee J.K., Kim Y.W., Kim B.T., Bae S.K. (2014). Selective inhibition of cytochrome P450 2D6 by Sarpogrelate and its active metabolite, M-1, in human liver microsomes. Drug Metab. Dispos..

[18] Zheng Y.F., Bae S.H., Choi E.J., Park J.B., Kim S.O., Jang M.J., Park G.H., Shin W.G., Oh E., Bae S.K. (2014). Evaluation of the in vitro/in vivo drug interaction potential of BST204, a purified dry extract of ginseng, and its four bioactive ginsenosides through cytochrome P450 inhibition/induction and UDP-glucuronosyltransferase inhibition. Food Chem. Toxicol..

[19] Li G., Huang K., Nikolic D., van Breemen R.B. (2015). High-throughput cytochrome P450 cocktail inhibition assay for assessing drug-drug and drug-botanical interactions. Drug Metab. Dispos..

[20] Kim H.J., Lee H., Ji H.K., Lee T., Liu K.H. (2019). Screening of ten cytochrome P450 enzyme activities with 12 probe substrates in human liver microsomes using cocktail incubation and liquid chromatography-tandem mass spectrometry. Biopharm. Drug Dispos..

[21] Valicherla G.R., Mishra A., Lenkalapelly S., Jillela B., Francis F.M., Rajagopalan L., Srivastava P. (2019). Investigation of the inhibition of eight major human cytochrome P450 isozymes by a probe substrate cocktail in vitro with emphasis on CYP2E1. Xenobiotica.

[22] Kumar V., Wahlstrom J.L., Rock D.A., Warren C.J., Gorman L.A., Tracy T.S. (2006). CYP2C9 inhibition: Impact of probe selection and pharmacogenetics on in vitro inhibition profiles. Drug Metab. Dispos..

[23] Yasar U., Tybring G., Hidestrand M., Oscarson M., Ingelman-Sundberg M., Dahl M.L., Eliasson E. (2001). Role of CYP2C9 polymorphism in losartan oxidation. Drug Metab. Dispos..

[24] Elsby R., Surry D.D., Smith V.N., Gray A.J. (2008). Validation and application of Caco-2 assays for the in vitro evaluation of development candidate drugs as substrates or inhibitors of P-glycoprotein to support regulatory submissions. Xenobiotica.

[25] Markowska M., Oberle R., Juzwin S., Hsu C.P., Gryszkiewicz M., Streeter A.J. (2001). Optimizing Caco-2 cell monolayers to increase throughput in drug intestinal absorption analysis. J. Pharmacol. Toxicol. Methods.

[26] Cho M.A., Yoon J.G., Kim V., Kim H., Lee R., Lee M.G., Kim D. (2019). Functional characterization of pharmcogenetic variants of human cytochrome P450 2C9 in Korean populations. Biomol. Ther. (Seoul).

[27] Yoo H., Ryu K.H., Bae S.K., Kim J. (2014). Simultaneous determination of trifolirhizin, (-)-maackiain, (-)-sophoranone, and 2-(2,4-dihydroxyphenyl)-5,6-methylenedioxybenzofuran from Sophora tonkinensis in rat plasma by liquid chromatography with tandem mass spectrometry and its application to a pharmacokinetic study. J. Sep. Sci..

[28] Bourrié M., Meunier V., Berger Y., Fabre G. (1996). Cytochrome P450 isoform inhibitors as a tool for the investigation of metabolic reactions catalyzed by human liver microsomes. J. Pharmacol. Exp. Ther..

[29] Obach R.S., Walsky R.L., Venkatakrishnan K. (2006). Mechanism-based inactivation of human cytochrome P450 enzymes and the prediction of drug-drug interactions. Drug Metab. Dispos..

[30] Parkinson A., Kazmi F., Buckley D.B., Yerino P., Paris B.L., Holsapple J., Toren P., Otradovec S.M., Ogilvie B.W. (2011). An evaluation of the dilution method for identifying metabolism-dependent inhibitors of cytochrome P450 enzymes. Drug Metab. Dispos..

[31] Stresser D.M., Mao J., Kenny J.R., Jones B.C., Grime K. (2014). Exploring concepts of in vitro time-dependent CYP inhibition assays. Expert Opin. Drug Metab. Toxicol..

[32] Xu L., Wang W. (2002). Herbs for clearing heat. Chinese Materia Medica: Combinations and Applications.

[33] US Food and Drug Administration (2005). Guidance for Industry: Estimating the Maximum Safe Starting Dose in Initial Clinical Trials for Therapeutics in Adult Healthy Volunteer. https://www.fda.gov/media/72309/download.

[34] Foti R.S., Rock D.A., Wienkers L.C., Wahlstrom J.L. (2010). Selection of alternative CYP3A4 probe substrates for clinical drug interaction studies using in vitro data and in vivo simulation. Drug Metab. Dispos..

[35] Van Booven D., Marsh S., McLeod H., Carrillo M.W., Sangkuhl K., Klein T.E., Altman R.B. (2010). Cytochrome P450 2C9-CYP2C9. Pharmacogenet. Genom..

[36] European Medicine Agency (2012). Guideline on the Investigation of Drug Interactions. https://www.ema.europa.eu/en/documents/scientific-guideline/guideline-investigation-drug-interactions-revision-1_en.pdf.

[37] US Food and Drug Administration (2020). Guidance for industry: In Vitro Drug Interaction Studies-Cytochrome P450 Enzyme-and Transporter-Mediated Drug Interactions. https://www.fda.gov/media/134582/download.

[38] Karjalainen M.J., Neuvonen P.J., Backman J.T. (2007). Tolfenamic acid is a potent CYP1A2 inhibitor in vitro but does not interact in vivo: Correction for protein binding is needed for data interpretation. Eur. J. Clin. Pharmacol..

[39] Jaakkola T., Backman J.T., Neuvonen M., Niemi M., Neuvonen P.J. (2006). Montelukast and zafirlukast do not affect the pharmacokinetics of the CYP2C8 substrate pioglitazone. Eur. J. Clin. Pharmacol..

[40] Kim K.A., Park P.W., Kim K.R., Park J.Y. (2006). Effect of multiple doses of montelukast on the pharmacokinetics of rosiglitazone, a CYP2C8 substrate, in humans. Br. J. Clin. Pharmacol..

[41] Walsky R.L., Obach R.S., Gaman E.A., Gleeson J.P., Proctor W.R. (2005). Selective inhibition of human cytochrome P4502C8 by montelukast. Drug Metab. Dispos..

[42] Press B., di Grandi D. (2008). Permeability for intestinal absorption: Caco-2 assay and related issues. Curr. Drug Metab..

