# Supplementary material for: Lack of Correlation between In Vitro and In Vivo Studies on the Inhibitory Effects of (‒)-Sophoranone on CYP2C9 Is Attributable to Low Oral Absorption and Extensive Plasma Protein Binding of (‒)-Sophoranone
Source: Pharmaceutics. 2020 Apr 7;12(4):328. doi: 10.3390/pharmaceutics12040328 (PMC7238241; doi:10.3390/pharmaceutics12040328)
Supplement: Supplementary file 1 [file pharmaceutics-12-00328-s001.zip › Supplemental Table 1.docx]

**Supplemental Table 1.** *K*_i_ values and inhibition types for CYP2C9 by SPN and sulfaphenazole in human liver microsomes (*n* = 3)

| **Inhibitors** | **CYP2C9 probe** | ***K_i_* values (μM)** | **R^2^** | **95% CI^a^ (μM)** | **Mode of inhibition^b^** |
| --- | --- | --- | --- | --- | --- |
| SPN | Diclofenac | 0.587 ± 0.0470 | 0.985 | 0.509‒0.664 | Competitive |
| SPN | Tolbutamide | 0.503 ± 0.0383 | 0.983 | 0.426‒0.580 | Competitive |
| Sulfaphenazole | Tolbutamide | 0.267 ± 0.0170 | 0.986 | 0.233‒0.301 | Competitive |

Data represent the mean ± standard deviation of triplicate.

Concentrations of SPN and sulfaphenazole were as following; 0–5 μM for SPN and 0–2 μM for sulfaphenazole, respectively.

^a^ Confidence interval

^b^ Inhibition type was determined by the best fit to competitive mode based on AIC values.
