# Supplementary figures and images for: Lack of Correlation between In Vitro and In Vivo Studies on the Inhibitory Effects of (‒)-Sophoranone on CYP2C9 Is Attributable to Low Oral Absorption and Extensive Plasma Protein Binding of (‒)-Sophoranone
Source: Pharmaceutics. 2020 Apr 7;12(4):328. doi: 10.3390/pharmaceutics12040328 (PMC7238241; doi:10.3390/pharmaceutics12040328)

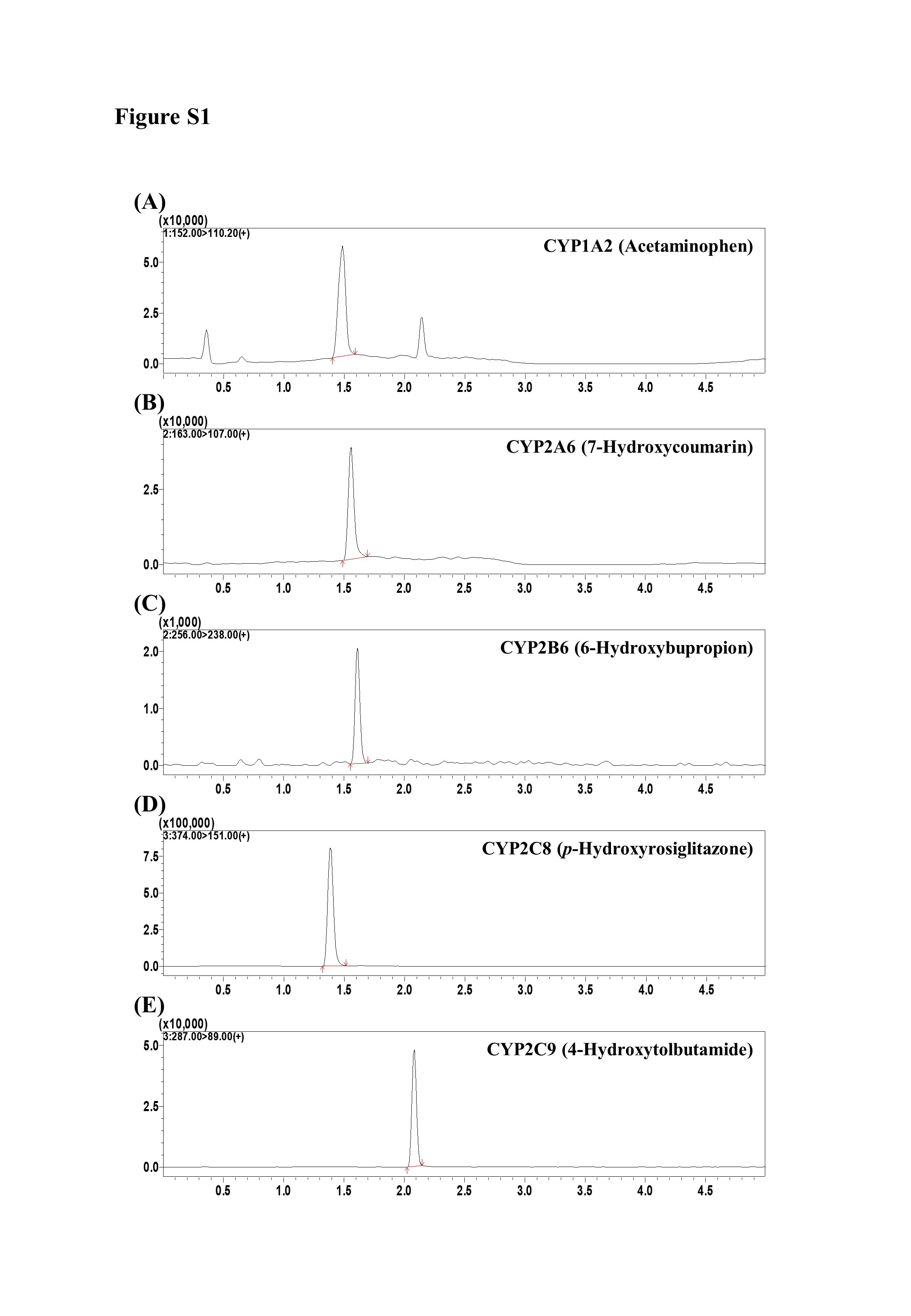

Supplement: Supplementary file 1 [file pharmaceutics-12-00328-s001.zip › Figure S1_1.JPG]

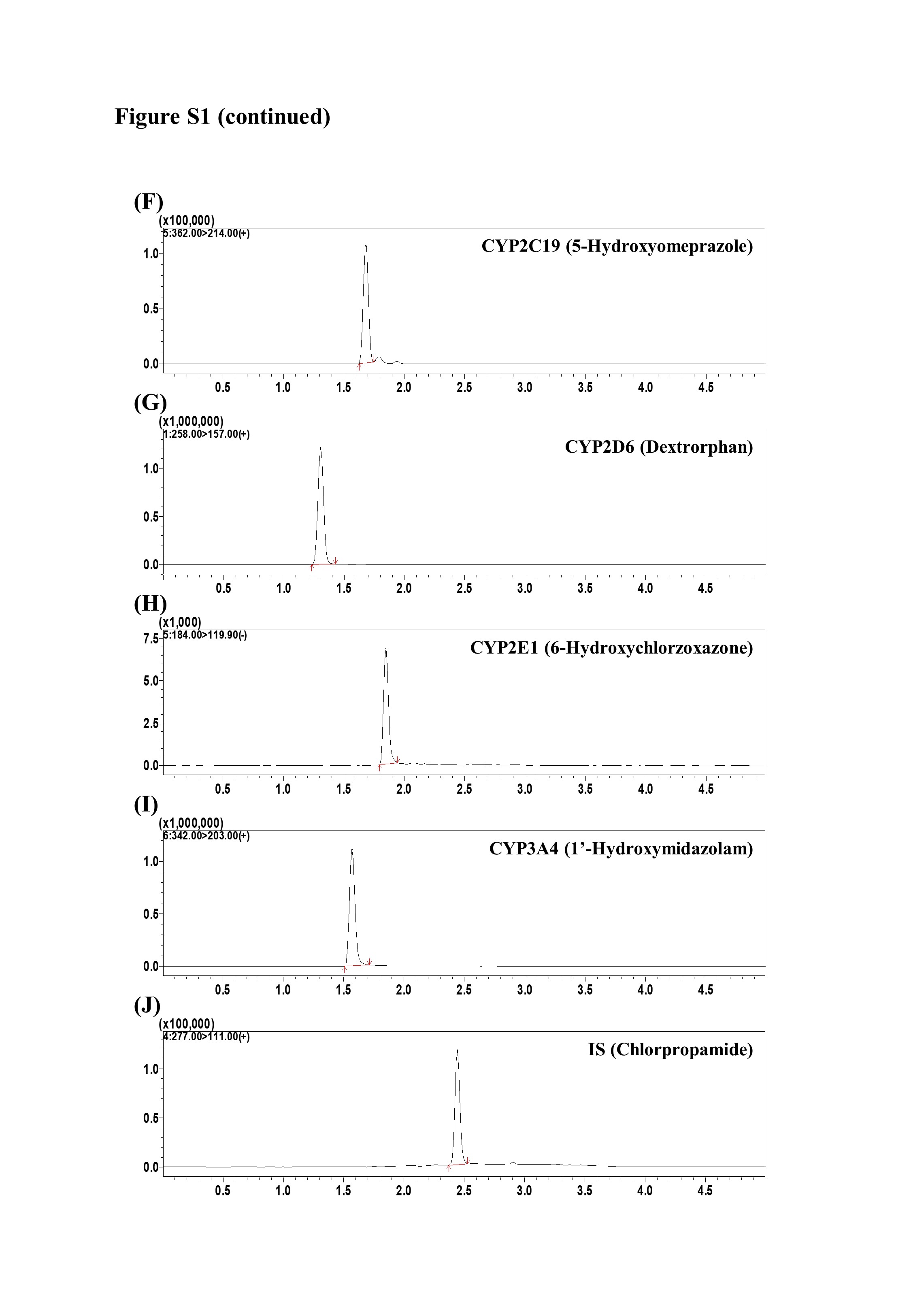

Supplement: Supplementary file 1 [file pharmaceutics-12-00328-s001.zip › Figure S1_2.JPG]
